# Enhanced Bioactivity of Quercetin–Tetrahydroisoquinoline Derivatives: Effect on Lipophilicity, Enzymes Inhibition, Antioxidant Potential, and Cytotoxicity

**DOI:** 10.3390/ijms252313076

**Published:** 2024-12-05

**Authors:** Marija Vučkovski, Ana Filipović, Milka Jadranin, Lela Korićanac, Jelena Žakula, Bojan P. Bondžić, Aleksandra M. Bondžić

**Affiliations:** 1 Vinča Institute of Nuclear Sciences, National Institute of the Republic of Serbia, University of Belgrade, P.O. Box 522, 11000 Belgrade, Serbia; marija.vuckovski@vin.bg.ac.rs (M.V.); lela@vin.bg.ac.rs (L.K.); pozegaj@vin.bg.ac.rs (J.Ž.); 2 University of Belgrade—Institute of Chemistry, Technology and Metallurgy, Department of Chemistry, Njegoševa 12, 11000 Belgrade, Serbia; ana.filipovic@nanosys.ihtm.bg.ac.rs (A.F.); milka.jadranin@ihtm.bg.ac.rs (M.J.)

**Keywords:** quercetin, tetrahydroisoquinolines, antioxidative activity, chelating ability, anticancer effect, Alzheimer’s disease

## Abstract

Quercetin, a well-known flavonoid with significant medicinal potential, was derivatized at the C8 position with a tetrahydroisoquinoline (THIQ) moiety, and physicochemical and pharmacological properties, inhibition potential, antioxidant activity, and cytotoxicity of new compounds were evaluated. Physicochemical and pharmacological properties, including lipophilicity, membrane permeability, and P-glycoprotein substrate affinity, were assessed theoretically using the SwissADME software. The metal-chelating ability of the new compounds was evaluated on metal ions Fe^2+^, Zn^2+^, and Cu^2+^, whose homeostasis disruption is linked to the development of Alzheimer’s disease. Inhibition potential was tested on the cholinergic enzymes acetylcholinesterase and butyrylcholinesterase, as well as Na^+^, K^+^-ATPase, an enzyme commonly overexpressed in tumours. Antioxidant potential was assessed using the DPPH assay. Cytotoxicity studies were conducted on healthy MRC-5 cells and three cancer cell lines: HeLa, MDA-231, and MDA-468. The results indicated that derivatization of quercetin with THIQ yielded compounds with lower toxicity, preserved chelating ability, improved antioxidant potential, increased selectivity toward the cholinergic enzyme butyrylcholinesterase, and enhanced inhibition potential toward Na^+^, K^+^-ATPase and butyrylcholinesterase compared to quercetin alone. Therefore, the synthesized derivatives represent compounds with an improved profile and could be promising candidates for further optimization in developing drugs for neurodegenerative and cancer diseases.

## 1. Introduction

Flavonoids are a diverse group of natural compounds known for their wide-ranging biological effects. Among them, quercetin (Q) stands out as one of the most studied and widely accessible. It is commonly found in various fruits, vegetables, leaves, and grains. In medicinal chemistry, quercetin has gained significant attention due to its broad pharmacological properties, including anti-neuroinflammation effect [1], cardiovascular protection [2], anticancer [3,4], antiulcer [5], antiallergic [6], antiviral [7], and anti-inflammatory effects [3]. These benefits are largely attributed to its strong antioxidant activity, which involves neutralizing free radicals and working in tandem with the body’s enzymes and natural antioxidants. An important part of this antioxidant action is quercetin’s ability to chelate transition metals like iron and copper, forming stable complexes that block these metals from participating in harmful free-radical production. By binding to these metals, chelating compounds can also alter their redox properties, rendering them inactive and reducing oxidative stress [8]. Additionally, quercetin’s versatility in medicinal chemistry is based on its ability to target multiple biological pathways through various molecular mechanisms, such as modulating signal transduction pathways and inhibiting specific enzymes involved in cancer cell survival. These induce apoptosis in cancer cells, inhibit cancer cell proliferation, and suppress tumour growth, making quercetin a compound of significant interest for drug design and development across various therapeutic areas. However, despite the promising therapeutic potential of quercetin, its use is hindered by several challenges, including low water solubility, limited bioavailability, rapid metabolism, instability, and difficulty in crossing the blood–brain barrier (BBB) [9,10].

Tetrahydroisoquinolines (THIQs) represent a versatile class of compounds with a broad spectrum of medical applications. These compounds exhibit anti-inflammatory properties, can act as ligands for central nervous system (CNS) receptors, and possess antitumour effects [11]. Furthermore, hybrid compounds incorporating a tetrahydroisoquinoline moiety have demonstrated strong antitumour effects [12], as well as neuroprotective and anti-Alzheimer properties [13]. Moreover, several drugs containing the THIQ moiety are already on the market for cancer treatment.

**Scheme 1:**
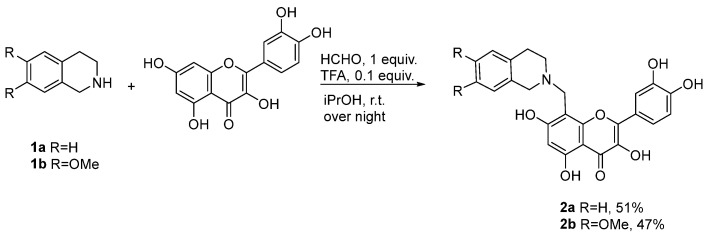
Synthesis of quercetin derivatives **2a** and **2b**.

The literature data indicate that derivatives of quercetin [14] as well as its isomers [15,16] may possess similar or, in some cases, improved antioxidative potential. Therefore, this study aims to investigate the impact of quercetin derivatization by THIQs on its physico-chemical and pharmacological profile, its chelating and antioxidative ability, its inhibition potential toward cholinergic enzymes and the ion pump, Na^+^, K^+^-ATPase, and its cytotoxicity. Initially, we synthesized new compounds by modifying quercetin at position 8 (the resorcinol ring) with 1,2,3,4-tetrahydroisoquinolines (compounds **1a** and **1b**, Scheme 1) following previously published procedure [17]. This was achieved through the Mannich reaction, involving quercetin and the iminium ion derived from formaldehyde and 1,2,3,4-tetrahydroisoquinolines, **1a** and **1b**. Subsequently, we assessed the influence of derivatization on the physicochemical and pharmacological profile using SwissADME software. The antioxidative potential was evaluated by the DPPH assay, while chelating ability was tested with Fe^2+^, Cu^2+^, and Zn^2+^ ions. The impact on enzyme activity was examined for enzymes involved in Alzheimer’s disease pathology, acetylcholinesterase (AChE) and butyrylcholinesterase (BuChE), as well as for the sodium pump, Na^+^, K^+^-ATPase, which is overexpressed in certain tumours and considered a potential target in antitumour drug development. Additionally, the toxicity profile was evaluated on a healthy cell line, MRC-5, while antitumour activity was assessed on three cancer cell lines: HeLa, MDA-MB-231, and MDA-MB-468.

## 2. Results and Discussion

### 2.1. Chemistry

Synthesis of C8-aminomethylated quercetin–1,2,3,4-tetrahydroisoquinoline derivatives was performed via the Mannich reaction of quercetin with iminium ions formed in situ from 1-phenyl-(or H)-6,7-dimethoxy-1,2,3,4-tetrahydroisoquinoline and formaldehyde, as shown in Scheme 1. Two monosubstituted quercetin derivatives **2a** and **2b** were synthesized in good yields.

### 2.2. Assessment of Drug-like Properties and Pharmacological Profiles of the Synthesized Quercetin–Tetrahydroisoquinoline Hybrids

As above mentioned, despite quercetin promising pharmacological properties, it faces several limitations in medicinal chemistry that impact its clinical applicability. One of the main challenges is its low bioavailability, meaning that only a small amount of ingested quercetin is absorbed and reaches systemic circulation. Additionally, low lipophilicity of the compound, expressed as a negative log *p* value, is the reason for its failure to penetrate the BBB, which has placed it among numerous other rejected compounds for AD treatment. In order to assess the effect of introducing tetrahydroisoquinoline motifs into the structure of quercetin on its physicochemical properties, the SwissADME software was used for theoretical predictions. The drug-likeness and pharmacokinetic profiles were predicted based on physicochemical properties of compounds, ability to cross the BBB, and whether they act as P-glycoprotein (P-gp) substrates. According to Lipinski’s rule, poor absorption and permeation are expected when a compound has a molecular weight greater than 500 Da, a lipophilicity (LogP) and hydrogen bond donors greater than 5, and hydrogen bond acceptors greater than 10. Additionally, logP values of synthesized derivates and their constituents were experimentally determined using the “shake flask” method.

According to the SwissADME prediction and the results obtained by the “shake flask” method, derivatization of quercetin with more lipophilic 1,2,3,4-tetrahydroisoquinolines or 6,7-dimethoxy-1,2,3,4-tetrahydroisoquinolines results in new compounds with significantly increased lipophilicity (Table 1). Furthermore, the new quercetin–tetrahydroisoquinoline hybrids satisfy Lipinski’s rule of five and do not act as P-gp substrates. However, despite improved lipophilicity and lack of interaction with P-gp, the main efflux transporter of the BBB, according to SwissADME predictions, these hybrids are still unable to cross the BBB. Experimentally determined log *p* values are presented in Table 1.

It is probable that future modifications of our compounds with more hydrophobic groups (such as fluorine or alkyl chains) could lead to higher lipophilicity and consequently facilitate easier crossing of the membrane [18]. However, a balance is necessary, as excessively lipophilic compounds may not be bioavailable or could have off-target effects. In our previously published paper [13], we demonstrated that certain C-1 derivatized THIQs were capable of crossing an artificial BBB. Based on these findings, we believe that combining them with quercetin could further enhance quercetin’s BBB-crossing potential.

### 2.3. Antioxidant Activity In Vitro

**Figure 1:**
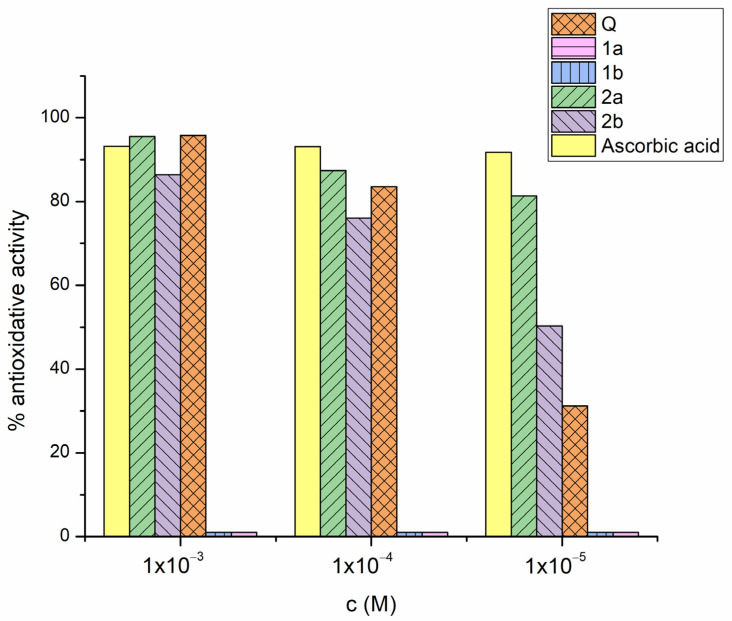
Antioxidative activity (%) of quercetin, Q; its tetrahydroisoquinoline derivates, **2a** and **2b**; ascorbic acid; 1,2,3,4-tetrahydroisoquinoline, **1a**; and 6,7-dimethoxy-1,2,3,4-tetrahydroisoquinoline, **1b**, toward DPPH radical at 0.01, 0.1, and 1mM concentration after 30 min of incubation.

Oxidative stress is considered one of the key factors contributing to the progression of various diseases, including cardiovascular diseases, diabetes, neurodegenerative disorders, and cancer [19,20]. Quercetin is well known for its strong antioxidative potential, which highlights its value in medical applications [21]. To evaluate the antioxidative potential of the new tetrahydroisoquinoline-based derivatives of quercetin, a DPPH assay was conducted with ascorbic acid as a positive control (Figure 1). 1,2,3,4-tetrahydroisoquinolines and 6,7-dimethoxy-1,2,3,4-tetrahydroisoquinolines, **1a** and **1b**, did not show antioxidative ability, while the experimentally obtained IC_50_ values for quercetin and ascorbic acid were 19.3 µM and 0.62 µM, respectively (Table 2).

Based on the obtained results, introduction of the tetrahydroisoquinoline moiety into quercetin structure improves its antioxidative potential. Enhanced antioxidative activity of synthesized derivates could be associated with the presence of the tertiary amino group in new compounds. Namely, delocalization of the nitrogen’s electron pair at the tertiary amino group combined with strong electron-donating phenolic OH groups at the catechol B-ring of quercetin [22] further boosts the antioxidant properties of our new derivates of quercetin [23]. On the other hand, the literature data show that the presence of multiple methoxy groups on the A-ring of flavones (especially those in the ortho position) can diminish the positive effects typically associated with a catechol group on the B-ring of quercetin [24]. Therefore, the approximately ten times better antioxidative activity of compound **2a** can likely be attributed to the absence of 6,7-dimethoxy groups on the tetrahydroisoquinoline motif.

### 2.4. Metal-Chelating Properties of Synthesized Quercetin Derivates

**Figure 2:**
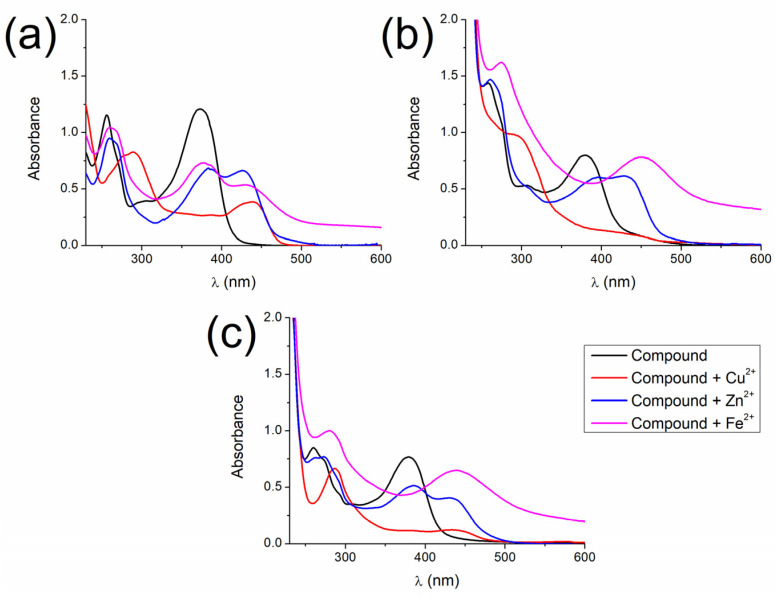
UV/Vis spectra of 5 × 10^−^^5^ M (**a**) Q, (**b**) **2a**, and (**c**) **2b** in the presence of different metal ions: Cu^2+^ (red), Zn^2+^ (blue), and Fe^2+^ (pink) at concentration 1 × 10^−^^4^ M, recorded 30 min after the addition of metal ions (t = 30 min).

It is well known that quercetin is able to chelate bio-metal ions such as Fe^2+^, Zn^2+^, and Cu^2+^. Disruption of the homeostasis of these metal ions, particularly Cu^2+^, is associated with the development and progression of AD by promoting the aggregation of Aβ (amyloid-β), which contributes to neurotoxicity in individuals with AD [25]. With this in mind, along with the neuroprotective ability of quercetin [26], the chelating ability of the new hybrid compounds (**2a** and **2b**) was also tested and compared to that of quercetin. The chelation ability was evaluated by comparing changes in the absorption spectra of quercetin and its derivatives, compounds **2a** and **2b**, following the addition of the aforementioned metal ions. The UV/Vis spectra of quercetin in ethanol show absorption maxima at 255 nm (I band) and 370 nm (II band) [27] (Figure 2a); as for its derivatives, compound **2a** exhibits absorption maxima at 260 nm (I band) and 378 nm (II band) (Figure 2b), and compound **2b** at 258 nm (I band) and 379 nm (II band) (Figure 2c). After the addition of the tested metal ions to quercetin–ethanol solution, bathochromic shifts (red shift) of both absorption maxima were observed with the appearance of new peaks. For Fe^2+^, the new peaks were observed at 273 nm and 438 nm [28], for Cu^2+^ at 293 nm and 436 nm [29,30], while after the addition of Zn^2+^, three new maxima were observed at 260, 382, and 438 nm [31]. Similarly, the red shift of absorption maxima was observed after the addition of metal ions in the solution of our derivatized compounds indicating the preserved chelating ability of the new hybrid compounds.

**Figure 3:**
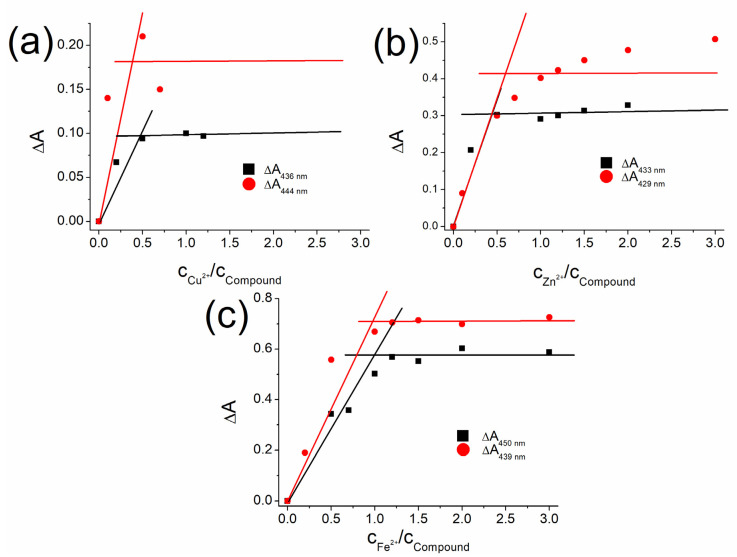
$∆A=f\left(c_{M^{2+}}{/c}_{\mathrm{compound}}\right)$ at absorption maxima of complexes for different metal ions: (**a**) Cu^2+^, (**b**) Zn^2+^, and (**c**) Fe^2+^; **2a**—black squares; **2b**—red circles.

Binding stoichiometry was determined using the molar ratio method, with increasing concentrations of metal ions (from 0 to 1.5 × 10^−4^ M) (Figures S1 and S2). Absorption spectra were collected 30 min after mixing. The change in absorption at the wavelength of maximum absorbance for the formed complex was plotted against the metal ion (M^2+^ = Cu^2+^, Zn^2+^, and Fe^2+^) to compound ratio (c_M_^2+^/c_compound_) and is presented in Figure 3. This graph allows the determination of the binding ratio between the newly synthesized compounds and metal ions. Analysis of the intersection of the curves at specific ratios suggests that the binding stoichiometry for both Cu^2+^ and Zn^2+^ is 2, indicating that one Cu^2+^ or Zn^2+^ ion coordinates with two **2a** or **2b** molecules. In contrast, the results for Fe^2+^ indicate that only one Fe^2+^ ion can bind to both compounds.

### 2.5. Influence of the New Quercetin Derivates on the Enzyme Activity

#### 2.5.1. The Influence on the Cholinergic Enzymes

In our previous papers, it was shown that tetrahydroisoquinoline derivatives possess inhibitory potential toward the cholinergic enzymes acetylcholine and butyrylcholine esterase [13]. Additionally, the literature data indicate that quercetin can inhibit both enzymes, with IC_50_ values in the micromolar range [32,33,34]. In order to evaluate how derivatization of quercetin by tetrahydroisoquinoline influences quercetin’s inhibitory potency toward cholinergic enzymes, the inhibitory potency of synthesized quercetin–tetrahydroisoquinoline hybrids (**2a** and **2b**) and their constituents (Q, **1a** and **1b**) was assessed by screening their activity at a concentration of 1 × 10^−4^ M (Table 3). The screening results indicated that derivatization of quercetin enhanced selectivity toward BuChE for both hybrids (**2a** and **2b**), with a twofold improvement in the inhibitory potency of compound **2a**. In contrast, the inhibitory potency toward AChE was significantly reduced, with compound **2b** showing only 15% inhibition at 1 × 10^−4^ M (Table 3). On the other hand, the tetrahydroisoquinoline constituents **1a** and **1b** did not exhibit significant activity in the screening test compared to quercetin. Further, IC_50_ values for quercetin and the quercetin–tetrahydroisoquinoline hybrids were determined and are presented in Table 3 and Figure S3. It is evident from the obtained results that derivatization of quercetin with tetrahydroisoquinoline leads to higher selectivity toward BuChE, offering a new class of selective BuChE inhibitors that may be important for the treatment of late-stage AD.

#### 2.5.2. The Influence on the Ion Pump—Na^+^, K^+^-ATPase

As mentioned in the literature, Na^+^, K^+^-ATPase is an enzyme crucial not only for maintaining Na^+^ and K^+^ ion gradients across cell membranes but also for regulating functions in cellular signalling pathways, contributing to cancer cell survival, proliferation, and apoptosis [37,38]. Cancer cells often express different isoforms of Na^+^, K^+^-ATPase compared to normal cells, making it a target of interest in cancer therapy. Moreover, Na^+^, K^+^-ATPase activity affects the epithelial–mesenchymal transition (EMT), a process that allows cancer cells to gain migratory and invasive properties, critical for metastasis. By modulating Na^+^, K^+^-ATPase, it is possible to influence the EMT process and thus impact cancer progression. This aspect is particularly promising in targeting aggressive cancers that exhibit high metastatic potential [37,38]. Therefore, the inhibitory activity of the synthesized derivates, **2a** and **2b**, was also tested against this enzyme. The obtained results indicated that the derivatization of quercetin by 6,7-dimethoxy-1,2,3,4-tetrahydroisoquinoline, compound **2b**, enhanced quercetin’s activity toward Na^+^, K^+^-ATPase, resulting in a 50-fold decrease in IC_50_ values compared to bare quercetin (Table 3, Figure S4). On the other hand, the derivatization of quercetin with 1,2,3,4-tetrahydroisoquinoline, **1a**, led to a suppression of inhibitor activity of compound **2a**. This suggests that the two methoxy substituents on the THIQ motif are involved in the interaction with Na^+^, K^+^-ATPase, contributing to enzyme inhibition. The low micromolar activity of compound **2b** toward Na^+^, K^+^-ATPase highlights potential anticancer activity of this compound.

### 2.6. Cytotoxicity

Quercetin has shown promise in anticancer therapy, as it can induce apoptosis in cancer cells, inhibit cancer cell proliferation, and suppress tumour growth. As discussed above, derivatization of quercetin with a tetrahydroisoquinoline motif altered its biological activity toward cholinergic enzymes and the Na^+^, K^+^-ATPase ion pump. To evaluate how this derivatization affects toxicity and cytotoxicity, the effects of the synthesized derivatives were studied in healthy MRC-5 cells and three tumour cell lines: HeLa, MDA-MB-231, and MDA-MB-468.

**Figure 4:**
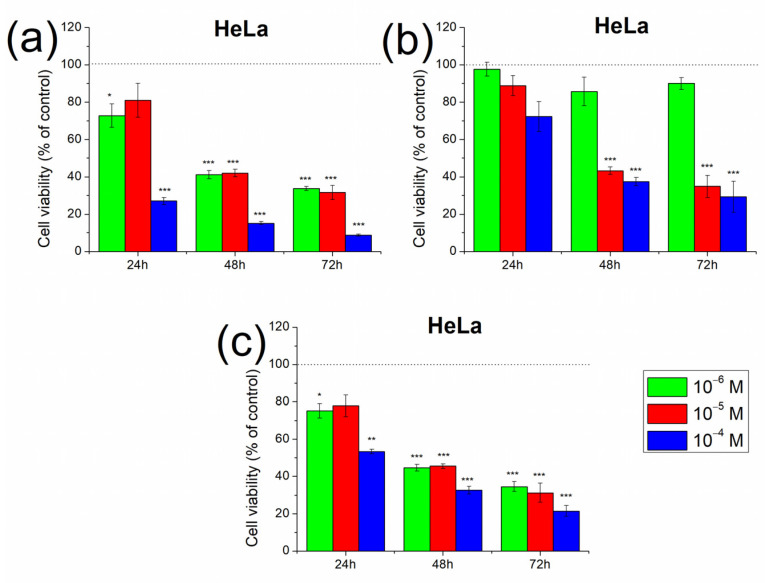
Viability of HeLa cells obtained by SRB assay 24, 48, and 72 h after treatment with quercetin (**a**), compound **2a** (**b**), and compound **2b** (**c**). Applied concentrations were 1 × 10^−6^, 1 × 10^−5^, and 1 × 10^−4^ M. Data obtained from four experiments are presented as mean ± S.D. Asterisks indicate statistical significance compared to the untreated control: * 0.01 < *p* < 0.05; ** 0.001 < *p* < 0.01; *** *p* < 0.001.

In vitro studies on healthy MRC-5 cells indicated that the synthesized derivatives have lower toxicity compared to quercetin, as demonstrated by their higher IC_50_ values than that of quercetin (Table 4). On the other hand, the tested cytotoxicity toward cancer cells revealed that the synthesized compounds exhibited time-, dose-, and cell-specific effects (Figure 4 and Figures S5–S7). An excellent inhibitory effect was observed in HeLa cells 48 and 72 h after treatment. At these time points, the compounds reduced cell viability to 43% (*p* < 0.001) and 37% (*p* < 0.001) after 48 h, and 35% (*p* < 0.001) and 29% (*p* < 0.001) after 72 h for compound **2a** (Figure 4). Compound **2b** generally showed a better inhibitory effect than compound **2a** in HeLa cells. Even the lowest concentration inhibited growth at all time points. Viability ranged from 53% to 78% (*p* < 0.05 for 1 × 10^−6^ M, *p* < 0.01 for 1 × 10^−4^ M) after 24 h, 32% to 45% (*p* < 0.001 for all concentrations) after 48 h, and 21% to 34% (*p* < 0.001 for all concentrations) after 72 h, compared to the corresponding control (Figure 4).

On the other hand, the decrease in the number of treated MDA-231 cells after 24 and 48 h was statistically insignificant. However, after 72 h, the viability of MDA-231 cells recovered to the control level or even increased for compound **2a** (Figure S6). No inhibition of MDA-468 cells by this compound was observed under the tested conditions (IC_50_ > 2 × 10^−4^ M) (Figure S7). In contrast to compound **2a**, compound **2b** significantly inhibited the growth of MDA-231 cells at the highest concentration, reducing their viability to 68–53% (*p* < 0.05 for 24 and 48 h, *p* < 0.01 for 72 h) (Figure S6). Additionally, compound **2b** showed a slightly better effect on MDA-468 cells than compound **2a** (Table 4), with the highest inhibition observed after treatment with the highest concentration for 72 h (56.56%, *p* < 0.001) (Figure S7). However, both synthesized compounds as well as quercetin did not selectively affect cancer versus healthy cells, as the selectivity index (SI) for all analysed cell lines was around or lower than 1. The selectivity could be increased by introducing more hydrophobic THIQs at the C8 position of Q, such as those previously developed in our group [13]. Lipophilic drugs can exhibit higher intracellular accumulation in cancer cells due to their altered membrane properties, leading to increased cytotoxicity specific to cancer cells while sparing normal cells [39]. Additionally, modification of these compounds with cancer-specific targets, such as HER2, EGFR, or folate receptors, can lead to preferential drug binding and entry into cancer cells [40].

Based on the cytotoxicity studies, it can be concluded that the synthesized compounds exhibit similar or lower toxicity in MRC-5 cells compared to quercetin, with similar cytotoxic effects in HeLa cells. On the other hand, improved cytotoxic effects were not observed in the MDA cell lines.

## 3. Materials and Methods

### 3.1. Chemistry

All reactions were monitored by thin-layer chromatography using Merck 60 F254 precoated silica gel plates (0.25 mm thickness). Preparative thin-layer chromatography was performed using Merck 60 F254 silica gel purchased from Merck KGA, Darmstadt, Germany.1H-NMR and 13C-NMR spectra were measured on a Bruker Ultrashield Advance III spectrometer (1H at 500 MHz, 13C at 125 MHz) and Varian 400 using DMSO-d6 as the solvent. Chemical shifts (δ) are given in parts per million (ppm) and coupling constants are given in hertz (Hz). The proton spectra are reported as follows δ/ppm (multiplicity, number of protons, and coupling constant, J/Hz). High-resolution mass spectrometry (HRMS) spectra were recorded only for new compounds using an Orbitrap Exploris 240 mass spectrometer (Thermo Fisher Scientific, Waltham, MA, USA), with heated electrospray ionization (HESI) as an ion source.

### 3.2. Chemicals and Reagents

Quercetin, MgCl_2_, ZnCl_2_, CuCl_2_ × 2H_2_O, FeSO_4_, NaCl, KCl, SnCl_2_, (NH_4_)_6_Mo_7_O_24_ × 4 H_2_O, tris(hydroxymethyl)aminomethane (TRIS), dimethyl sulfoxide (DMSO), ethanol, methanol, glycerol, sulfuric acid, acetylcholinesterase from electric eel (AChE), butyrylcholinesterase from equine serum (BuChE), Na/K-ATPase from the porcine cerebral cortex, adenosine triphosphate (ATP), acetylthiocholine iodide (AChI), butyrylthiocholine iodide (BuChI), 5,5′-Dithiobis (2-nitrobenzoic acid) (DTNB), sodium dodecyl sulfate (SDS), and 2,2-diphenyl-1-picrylhydrazyl (DPPH) were obtained from Sigma-Aldrich, Steinheim, Germany. Tetrahydroisoquinoline-modified quercetin compounds (**2a** and **2b**) were synthesized according to a previously published procedure [17].

### 3.3. ADME Prediction

The freely available software SwissADME (http://www.swissadme.ch, accessed on 1 October 2024) was used for in silico prediction of pharmacokinetic data and physical–chemical properties such as lipophilicity and the possibility of the compound passing through the BBB [41].

### 3.4. The Lipophilicity

To measure the lipophilicity (expressed as the partition coefficient) of the synthesized compounds and their constituents, using a “shake-flask” method, a two-phase system composed of n-octanol and deionized water was used. The stock solutions of the compounds were diluted with water to the concentration of 1 × 10^−4^ M, and this solution was used for further investigation. Next, 3 mL of this diluted stock solution was mixed with 3 mL of n-octanol and shaken using a mechanical shaker at 25 ± 1 °C (Orbital Shaker Incubator ES-20, Grant-bio) for 30 min at 250 rpm, centrifuged (Centrifuge 5702, Eppendorf, Hamburg, Germany) at 3000 rpm for 20 min to afford complete phase separation, and the n-octanol phase was removed. The absorbance of the aqueous phase was measured spectrophotometrically at 260 nm for Q and compounds **1a**, **1b**, **2a**, and **2b**. The concentration of the compounds was calculated from their extinction coefficient at 260 nm that was previously determined.

### 3.5. 2,2-Diphenyl-1-Picrylhydrazyl Radical Scavenging Assay

DPPH (2,2-diphenyl-1-picrylhydrazyl) radical scavenging assay was used to assess free-radical scavenging activity of synthesized compounds. Initially, 1 × 10^−4^ M working solution of DPPH was prepared, and 350 µL was added to 350 µL of the solution of each compound. The final concentrations of the compounds (Q, **2a**, and **2b**) in the samples were 1 × 10^−3^ M, 1 × 10^−4^ M, and 1 × 10^−5^ M. The control included ethanol only and DPPH–ethanol solution without compounds. The mixtures were incubated at 25 °C in the dark for 30 min. After incubation, the absorbance of the samples at 517 nm was measured in duplicate by using a UV/Vis spectrophotometer (Lambda 35, Perkin Elmer, Waltham, MA, USA). The DPPH radical scavenging ability was calculated using the following equation:

(1) $$\mathrm{Antioxidative}\;\mathrm{activity}(\%)=\left(\frac{A_{\mathrm{control}}{-A}_{\mathrm{sample}}}{A_{\mathrm{control}}}\right)\times 100\%$$

where A_sample_ and A_control_ represent the absorbance at 517 nm of the sample and control, respectively. All samples were analysed in duplicate.

### 3.6. Metal-Chelating Assay

The metal-chelating ability of synthesized compounds in ethanol was assessed using UV/Vis spectrophotometer (Lambda 35, Perkin Elmer) recording spectra over a wavelength range of 230–600 nm in a 1 cm quartz cell. The mixture of compound at 5 × 10^−5^ M of final concentration and metal ions at 1 × 10^−4^ M of final concentration was incubated for 30 min at room temperature. The tested temperature was 25 °C. Each sample was performed in triplicate.

The binding stoichiometry was assessed by adding increasing concentrations of metal ions (0–1.5 × 10^−4^ M) to a fixed concentration of compound (5 × 10^−5^ M) and incubating the mixture for 30 min at room temperature. The spectra were then measured as described above, and the ligand-to-metal chelation ratio in the complex was calculated.

### 3.7. AChE/BuChE Assay

The abilities of compounds to inhibit AChE and BuChE activity were determined following Ellman’s procedure, with AChI and BuChI as the enzyme substrate, respectively, and DTNB as a chromogenic reagent [42]. AChE’s and BuChE’s working solutions were prepared at 20 mM TRIS and deionized water, respectively. AChI and BuChI solutions were prepared in deionized water. A 0.01 M DTNB solution was prepared with 0.1 M phosphate buffer (pH 7) containing 0.15% (*w*/*v*) sodium bicarbonate. Stock solutions (10 mM) of investigated compounds were prepared daily by dissolving solid compounds in DMSO. The reaction mixture containing phosphate buffer, DTNB, enzyme, and compound was preincubated 30 min at 37 °C. After the preincubation time, the enzyme reaction was initiated by adding appropriate substrate and left to incubate for 6 min. After incubation, 0.1 M SDS was added to stop the reaction. Absorbance measurements were performed using a UV/Vis spectrophotometer (Lambda 35, Perkin Elmer), measuring absorbance at 412 nm. A blank assay, containing all components except the enzyme, was used to account for non-enzyme reactions. The control solution contained all components except the inhibitor. All experiments were conducted in triplicate, and the results are expressed as the mean percentage enzyme activity compared to the control value. IC_50_ values were determined graphically using Origin 9 Microsoft Windows.

### 3.8. Na^+^, K^+^-ATPase Assay

Na^+^, K^+^-ATPase activity was assessed using a modified version of the method by Seals et al. [43], based on measuring the change in orthophosphate concentration released during ATP hydrolysis. The incubation mixture was prepared at 50 mM TRIS-HCl (pH 7.4), containing 5 mM MgCl_2_, 100 mM NaCl, 20 mM KCl, 25 μL of 5mg/mL commercial enzyme solution, and 20 μL of an inhibitor solution at a certain concentration. After preincubating the mixture for 10 min at 37 °C, the reaction was initiated by adding 20 μL of 20 mM ATP. The reaction proceeded for 10 min at 37 °C before being terminated by adding 22 μL of 3 M cold perchloric acid, with the tube then placed on ice. In the blank sample, the incubation mixture contained only TRIS buffer (pH 7.4) without MgCl_2_, NaCl, or KCl. The reaction volume was adjusted to a final volume of 200 μL, after which 4.5 mL of water was added. Released orthophosphate was quantified using a colorimetric reaction with 200 μL of 0.2 M ammonium molybdate in 30% sulfuric acid and one drop of 2.5% SnCl_2_ in glycerol. Total ATPase activity was determined by measuring the absorbance of solution at 690 nm. Enzyme activity was expressed as relative enzyme activity (REA), defined as the percentage of Na^+^, K^+^-ATPase activity remaining in comparison to samples without inhibitor (control activity).

### 3.9. Cell Culture

Two human breast cancer cell lines, MDA-MB-231 (HTB-26) and MDA-MB-468 (HTB-132), human cervical cancer cell line HeLa (CCL-2), and MRC-5 (CCL-171) fibroblasts were purchased from the American Type Culture Collection (ATCC, Manassas, VA, USA). All cell lines were cultured in Dulbecco’s Modified Eagle’s Medium (DMEM) with 4500 mg/L glucose and l-glutamine (Capricorn Scientific, Ebsdorfergrund, Germany), supplemented with 10% foetal bovine serum (PAN-Biotech, Aidenbach, Germany), 10,000 U/mL penicillin, and 10 mg/mL streptomycin solution (Capricorn Scientific) under standard conditions at 37 °C in a humidified 5% CO_2_ atmosphere (ESCO, Lifesciences Group, Singapore).

### 3.10. SRB Assay

Cells were seeded on a 96-well plate (Thermo Fisher Scientific, Waltham, MA, USA) at 2 × 10^3^ cells per well in DMEM medium. For the analysis of the concentration- and time-dependent effect of the analysed compounds, cell monolayers were treated 24 h after seeding with concentrations 1 × 10^−6^, 1 × 10^−5^, and 1 × 10^−4^ M for 24, 48, or 72 h. Cell viability was detected using sulforhodamine B (SRB) assay, based on the staining of cellular proteins. The assay was performed according to the procedure described by Skehan et al. [44]. The cells were fixed with 10% trichloroacetic acid (TCA, CARLO ERBA Reagents GmbH, Emmendingen, Germany) for 1 h at 4 °C, washed with water, and stained for 15 min with 0.4% SRB (Sigma-Aldrich Chemie GmbH, Steinheim, Germany) in 1% acetic acid. The unbound SRB dye was rinsed with 1% acetic acid, while the protein-bound dye was extracted with a 10 mM Tris base (Sigma-Aldrich). The absorbance was measured at 550 nm with a reference wavelength of 690 nm in a microplate reader (Wallac, VICTOR2 1420 Multilabel counter, PerkinElmer, Turku, Finland). The results were expressed as a percentage of the control, where the cell growth of the control is set to 100%. IC_50_, defined as the concentration that caused a 50% loss of cell growth, was calculated by non-linear regression analysis with the ED_50_plus v1.0 software. The selectivity index (SI) was determined according to the following formula:

(2) $$\mathrm{SI}=\frac{{\mathrm{IC}}_{50}\;\mathrm{for}\;\mathrm{fibroblast}\;\mathrm{cell}\;\mathrm{line}}{{\mathrm{IC}}_{50}\;\mathrm{for}\;\mathrm{tumour}\;\mathrm{cell}\;\mathrm{line}}$$

### 3.11. Statistical Analysis

The assay was performed two times in 4 replicates for every experimental group. The statistical significance of differences between treated and untreated cells was estimated by the independent Student’s *t*-test, with the level of significance set at *p* < 0.05.

## 4. Conclusions

In conclusion, derivatization of quercetin at the C8 position (A ring) with a THIQ moiety can enhance its lipophilicity and chelating ability toward metal ions implicated in the pathogenesis of AD. Regarding inhibitory potential, it was observed that THIQ derivatization improves inhibition of Na^+^, K^+^-ATPase and BuChE and increases selectivity toward BuChE. Additionally, antioxidant testing indicated an increase in the antioxidative potential of the new compounds compared to quercetin alone. In terms of toxicity, the new compounds demonstrated lower toxicity in healthy MRC-5 cells and showed a similar cytotoxicity profile on HeLa cells. Therefore, the synthesized derivatives represent compounds with an improved profile and could be valuable candidates for further optimization in developing drugs for neurodegenerative and cancer diseases.

## Figures and Tables

**Table 1:** Predicted drug-likeness parameters (ADME test) for synthesized quercetin tetrahydroisoquinoline hybrids obtained using SwissADME.

Compound	Structure	SwissADME							Experimentally Obtained Results
		M, Da	H-Bond Donor	H-Bond Acceptor	logP	P-gp Substrate	BBB	Lipinski	logP
**1a**	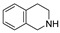	133.19	1	1	1.80	No	No	Yes	1.40
**1b**	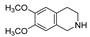	193.24	1	3	1.12	Yes	Yes	Yes	1.14
Quercetin	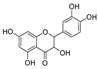	304.25	5	7	−0.64	No	No	Yes	1.60
**2a**	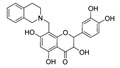	449.15	5	8	0.61	No	No	Yes	3.07
**2b**	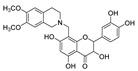	509.50	5	10	0.01	No	No	Yes	2.47

**Table 2:** Experimentally determined IC_50_ values for evaluated antioxidant DPPH assay of quercetin, Q; two new synthesized derivates, **2a** and **2b**; ascorbic acid; 1,2,3,4-tetrahydroisoquinoline, **1a**; and 6,7-dimethoxy-1,2,3,4-tetrahydroisoquinoline, **1b**.

Compound	DPPH, IC_50_, μM
Quercetin	19.3
**1a**	n.a.
**1b**	n.a.
**2a**	1.59
**2b**	10.2
Ascorbic acid	0.62

**Table 3:** The inhibition properties of investigated compounds toward *ee*AChE, *eq*BuChE, and Na^+^, K^+^-ATPase from pork cerebral cortex. The preincubation and incubation time were 6 min, the temperature was 37 °C.

Compound	Structure	Percentage Inhibition at 1 × 10^−4^ M			IC_50_, μM		
		AChE	BuChE	Na^+^, K^+^-ATPase	AChE	BChE	Na^+^, K^+^-ATPase
**1a**	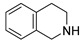	2	2	n.d.	n.d.	n.d.	25,000 ± 1700 [35]
**1b**	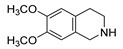	14.1	17.7	n.d.	n.d.	n.d.	n.d.
Quercetin	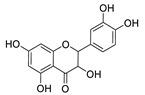	58	72	n.d.	67.8	22.1	46.32 [36]
**2a**	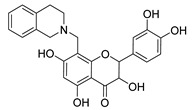	33.3	70	>100	n.d.	16.9	n.a.
**2b**	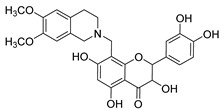	15.4	67.6	100	n.d.	41.1	0.97

n.a.—no activity; n.d.—not determined.

**Table 4:** In vitro cytotoxic activity given as IC_50_ in µM, calculated 72 h after treatment, for Q and its derivates **2a** and **2b**.

	IC_50_, µM			
	MRC	HeLa	MDA-231	MDA-468
**2a**	55.67 ± 2.78	53.50 ± 2.67	146 ± 7	>200
**2b**	16.60 ± 0.83	19.50 ± 0.97	106 ± 5	109 ± 5
Q	10.40 ± 0.52	15.10 ± 2.67	76.6 ± 3.8	65.9 ± 3.3

## Data Availability

Data are contained within the article and Supplementary Materials.

## Supplementary Materials

The following supporting information can be downloaded at: https://www.mdpi.com/1422-0067/25/23/13076/s1.
