# Supplementary material for: Enhanced Bioactivity of Quercetin–Tetrahydroisoquinoline Derivatives: Effect on Lipophilicity, Enzymes Inhibition, Antioxidant Potential, and Cytotoxicity
Source: Int J Mol Sci. 2024 Dec 5;25(23):13076. doi: 10.3390/ijms252313076 (PMC12068008; doi:10.3390/ijms252313076)
Supplement: Supplementary file 1 [file ijms-25-13076-s001.zip › ijms-3335876-supplementary.pdf]

# Enhanced Bioactivity of Quercetin-Tetrahydroisoquinoline Derivatives: Effect on Lipophilicity, Enzymes Inhibition, Antioxidant Potential, and Cytotoxicity

Marija Vučkovski [1], Ana Filipović [2], Milka Jadranin[2], Lela Korićanac[1], Jelena Žakula[1], Bojan P. Bondžić *[2] and Aleksandra M. Bondžić*[1]

*[1] Vinča Institute of Nuclear Sciences, National Institute of the Republic of Serbia, University of Belgrade, P.O. Box 522, 11000 Belgrade, Serbia. (aleksandrab@vin.bg.ac.rs)*
*[2] University of Belgrade-Institute of Chemistry, Technology and Metallurgy, National Institute of the Republic of Serbia, Njegoševa 12, 11000 Belgrade, Serbia.*

**Coresponding authors: aleksandrab@vin.bg.ac.rs; bojan.bondzic@ihtm.bg.ac.rs*

1.1. *Chemistry*

**General procedure for the synthesis of the Quercetin-THIQ derivatives**
Procedure adopted from [1] was followed. A solution of 1,2,3,4-tetrahydroisoquinoline (0.5 mmol) in i-PrOH (10 mL) was treated dropwise with a solution of QU (0.5 mmol) in i-PrOH (5 mL) with stirring over 10 min. The reaction mixture was treated dropwise with a solution of formaldehyde (32%, 0.5 mmol). The reaction mixture was stirred overnight. The course of the reaction was monitored by thin layer chromatography. The precipitate was separated and rinsed with i-PrOH. Purity of the precipitate was confirmed by analytical HPLC, and precipitate was purified using preparative TLC plates and preparative HPLC for analytically pure samples.[1]

**2-(3,4-Dihydroxyphenyl)-8-[(1,2,3,4-tetrahydroisoquinolin-2-yl)methyl]-3,5,7-trihydroxy-4Hchromen-4-one (2a).**

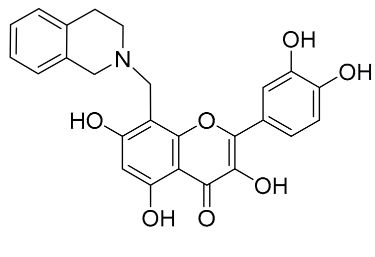


General procedure was followed staring from 1,2,3,4-tetrahydroisoquinoline, to give the title compound (105.5 mg, 47 %) as yellow solid. m.p. 206-210 °C.
**1H NMR** (DMSO-d6 + $CCl_4$, 500 MHz): 12.4 (1H, br.s.), 8.96 (1H, m), 8.33 (1H, m), 7.74 (1H, d, J = 2.3 Hz), 7.50 (1H, m), 7.30-7.20 (m, 2H), 6.75 (1H, d, *J* = 8.5 Hz), 6.56 (1H, s), 6.50 (1H, s), 6.11 (1H, s), 4.10 (2H, s), 3.82 (2H, s), 2.95 (2H, t, J = 5.9 Hz), 2.84 (2H, t, J = 5.9 Hz); **13C NMR** (DMSO-d6 + CCl4, 125 MHz): 177.41, 165.77, 162.51, 153.02, 149.14, 146.96, 146.52, 140.21, 126.11, 126.03, 125.88, 125.52, 123.44, 121.18, 117.01, 116.11, 113.32, 111.51, 105.03, 100.41, 99.70, 57.81, 53.22, 51.41, 27.33; **HRMS**: m/z (HESI) calc for $C_{25}H_{22}NO_7$ $[M+H]^+$ 448.1391, found 448.1390.

**2-(3,4-Dihydroxyphenyl)-8-[(6,7-dimethoxy-1,2,3,4-tetrahydroisoquinolin-2-yl)methyl]-3,5,7-trihydroxy-4Hchromen-4-one (2b).**

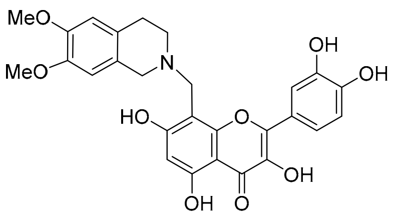


General procedure was followed staring from 6,7-dimethoxy-1,2,3,4-tetrahydroisoquinoline, to give the title compound (155 mg, 61 %) as yellow solid. m.p. 210-214 °C

**1H NMR** (DMSO-d6 + $CCl_4$, 500 MHz): 11.9 (1H, br.s.), 8.93 (1H, m), 8.24 (1H, m), 7.66 (1H, d, J = 2.1 Hz), 7.43 (1H, dd, J = 2.2, 8.3 Hz), 6.75 (1H, d, *J* = 8.5 Hz), 6.56 (1H, s), 6.50 (1H, s), 6.01 (1H, s), 4.02 (2H, s), 3.71 (3H, s), 3.69 (2H, s), 3.66 (3H, s), 2.89 (2H, t, J = 5.9 Hz), 2.78 (2H, t, J = 6.01 Hz); **13C NMR** (DMSO-d6 + CCl4, 125 MHz): 176.41, 164.82, 160.57, 154.04, 148.17, 148.03, 147.84, 146.84, 145.40, 136.29, 125.58, 125.44, 122.8, 120.08, 115.93, 115.58, 112.22, 110.41, 103.54, 99.39, 98.80, 55.86, 55.84, 54.98, 52.34, 50.45, 28.46. Spectroscopic data are in agreement with the published data. [1]

1.2.*Metal chelating properties of synthesized quercetin derivates*

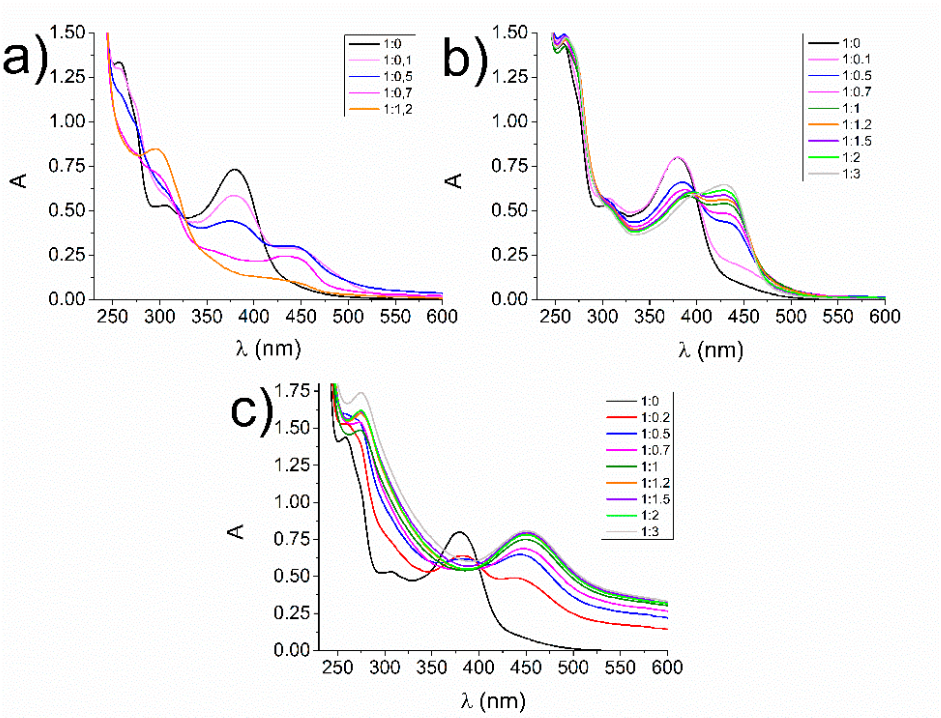


**Figure S1.** The UV/Vis spectra of 5 × $10^{-5}$ M **2a** in the presence of different metal ions **a)** $Cu^{2+}$, **b)** $Zn^{2+}$, **c)** $Fe^{2+}$ recorded 30 minutes after mixing **2a** and metal ion solutions. The **2a** to metal ion ratios were varied from 1:0 to 1:3 corresponding to metal ion concentrations ranging from 0 to 1.5 × $10^{-4}$ M.

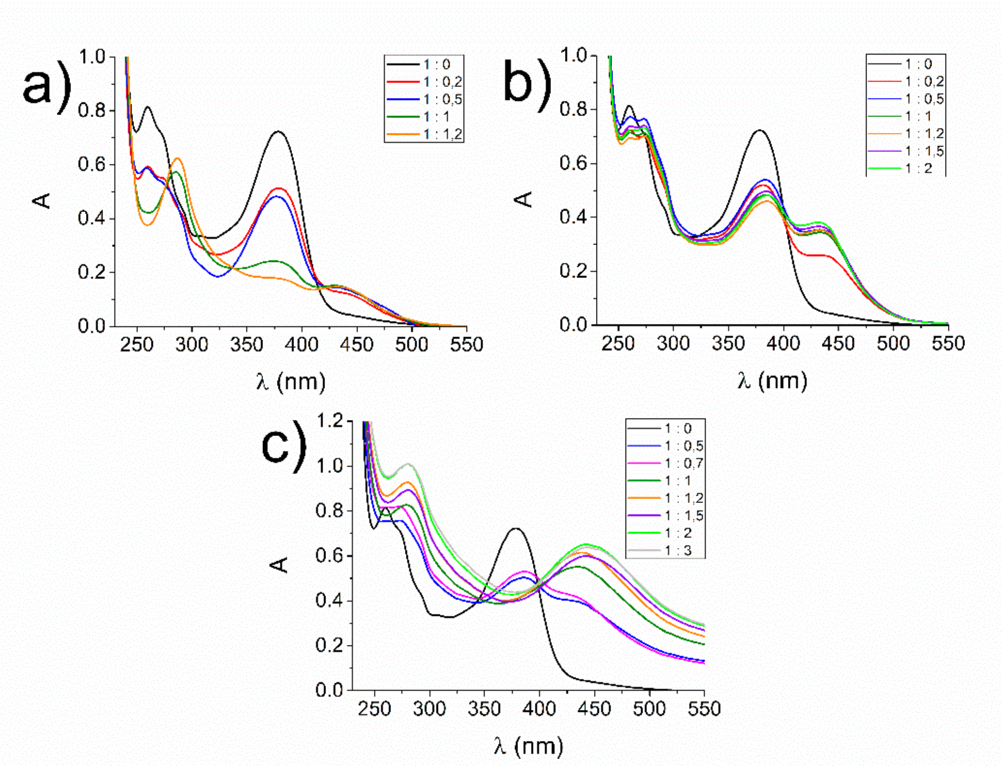


**Figure S2.** The UV/Vis spectra of 5 × 10$^{-5}$ M **2b** in the presence of different metal ions **a)** $Cu^{2+}$, **b)** $Zn^{2+}$, **c)** $Fe^{2+}$ recorded 30 minutes after mixing **2b** and metal ion solutions. The **2b** to metal ion ratios were varied from 1:0 to 1:3 corresponding to metal ion concentrations ranging from 0 to 1.5 × 10$^{-4}$ M.

1.3. *Influence of new quercetin derivates on the enzyme activity*

*1.3.1. The influence on the cholinergic enzymes*

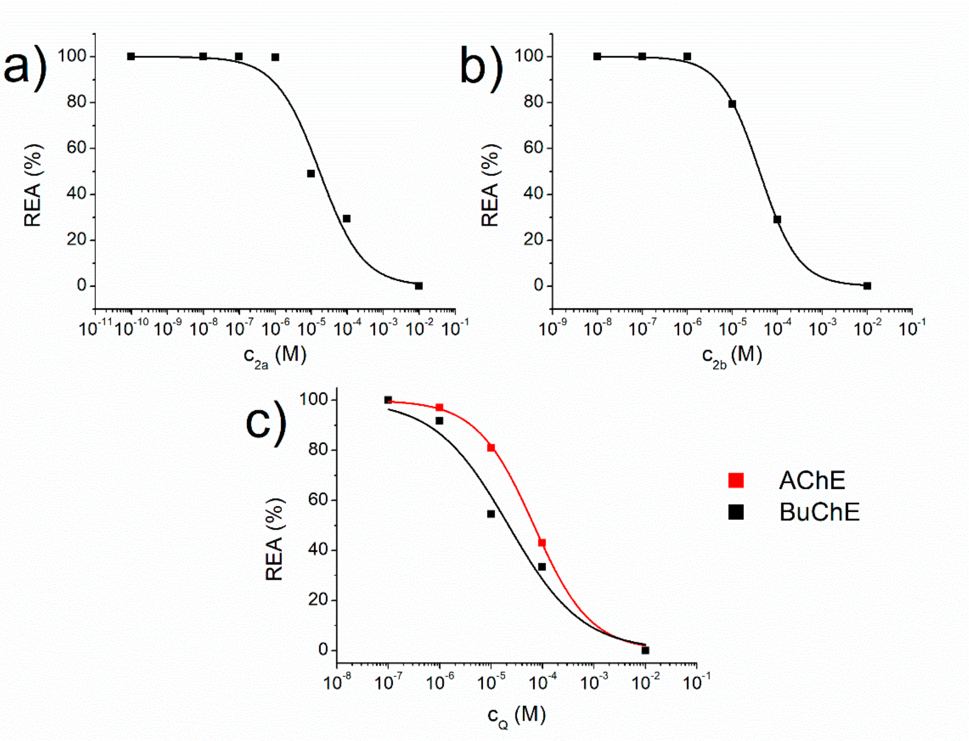


**Figure S3.** Inhibition of AChE (red squares) and BuChE (black squares) by compounds a) **2a**, b) **2b** and c) **Quercetin**.

*1.3.2. The influence on the ion pump – $Na^+$, $K^+$-ATPase*

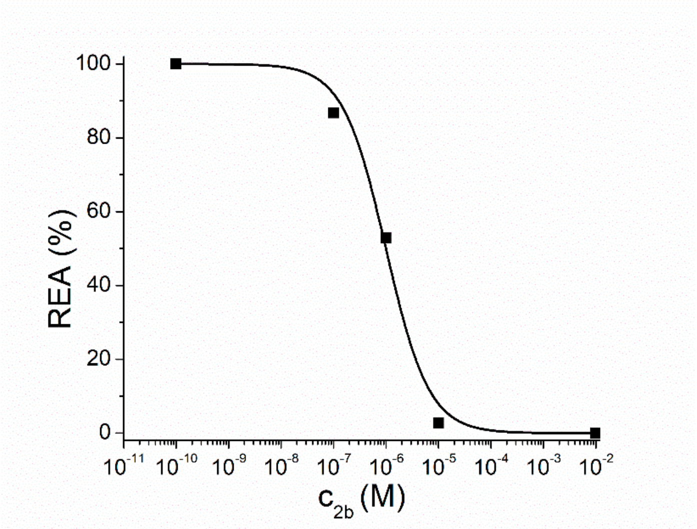


**Figure S4.** Inhibition of $Na^+$, $K^+$-ATPase (black squares) by the compound **2b**

1.4. *Cytotoxicity*

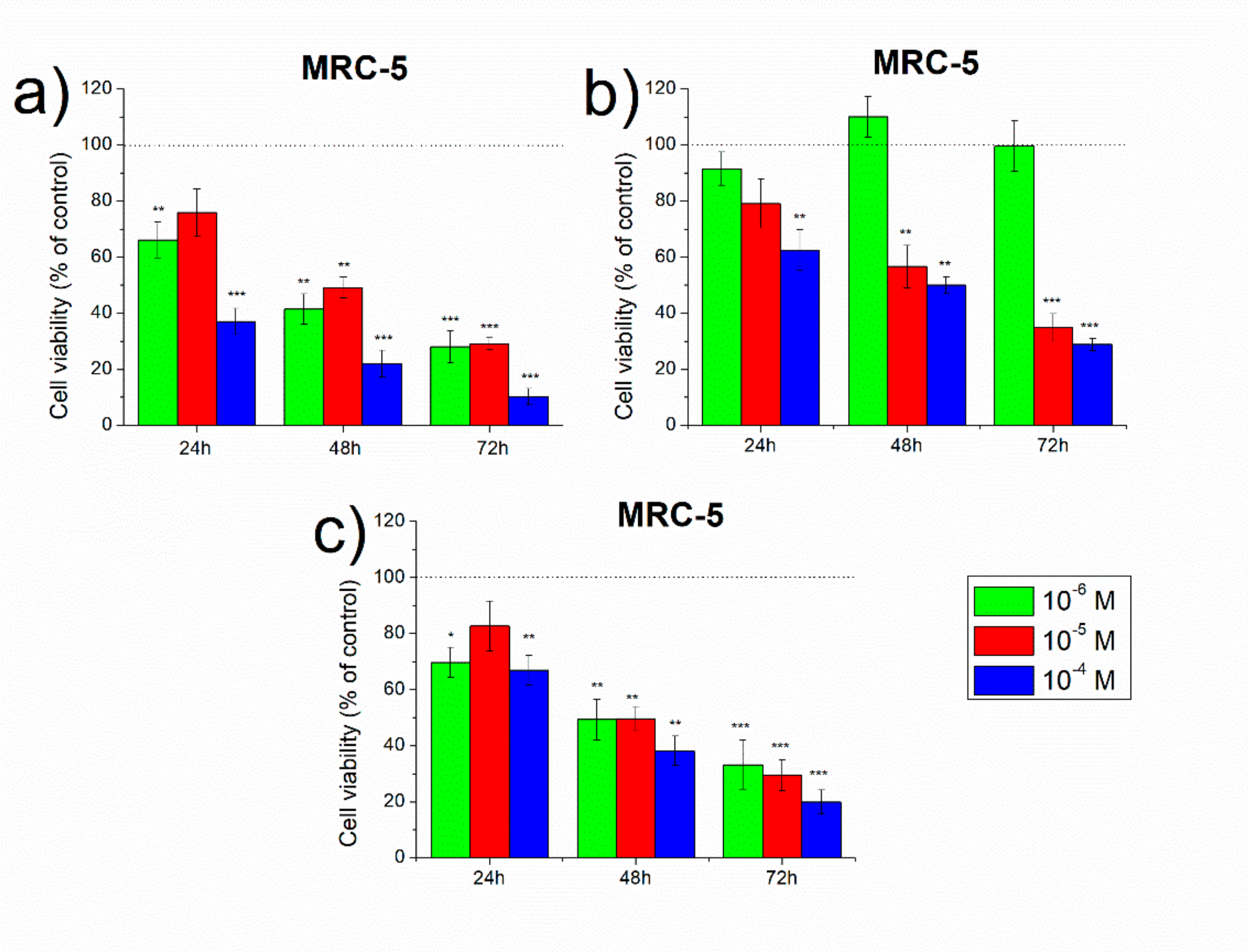


**Figure S5.** Viability of MRC-5 cells obtained by SRB assay, 24, 48, and 72 h after treatment with quercetin (A), the compound **2a** (B), and the compound **2b** (C). Applied concentrations were from 1, 10, and 100 μM. Data obtained from four experiments are presented as Mean ± S.D. * - Statistical significance compared to the untreated control; * - 0.01 $<p < 0.05$; ** - 0.001 $<p < 0.01$; *** - $p < 0.001$.

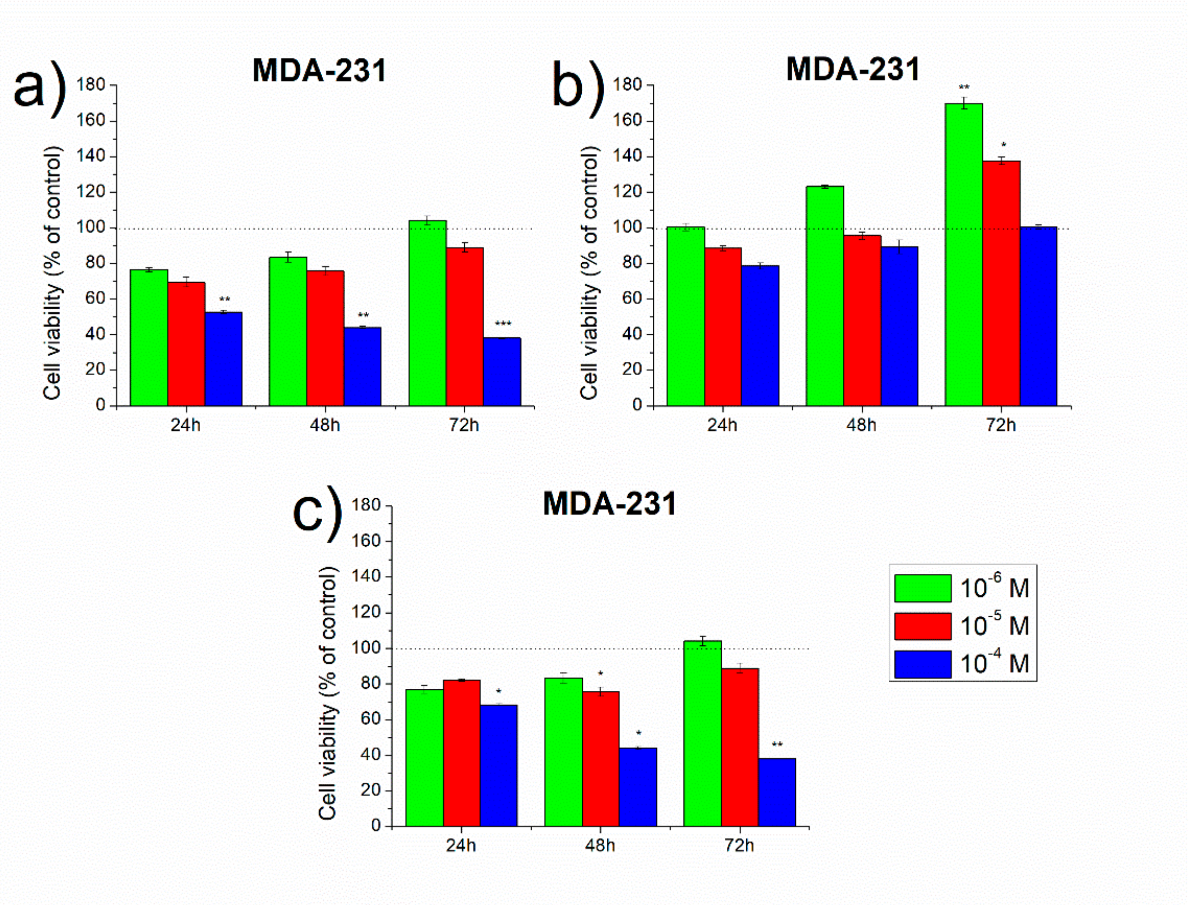


**Figure S6.** Viability of MDA-231 cells obtained by SRB assay, 24, 48, and 72 h after treatment with quercetin (A), the compound **2a** (B), and the compound **2b** (C). Applied concentrations were from 1, 10, and 100 µM. Data obtained from four experiments are presented as Mean ± S.D. * - Statistical significance compared to the untreated control; * - $0.01 < p < 0.05$; ** - $0.001 < p < 0.01$; *** - $p < 0.001$.

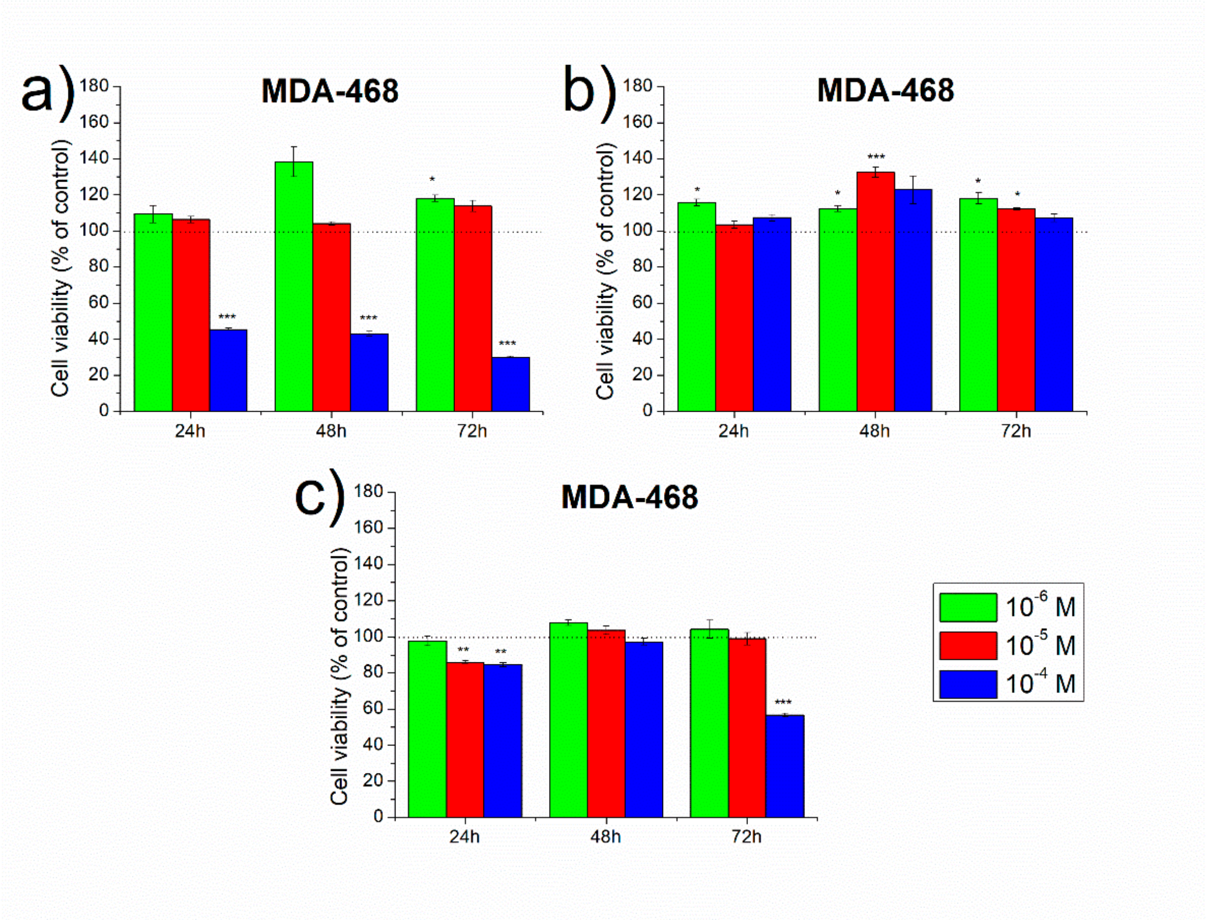


**Figure S7.** Viability of MDA-468 cells obtained by SRB assay, 24, 48, and 72 h after treatment with quercetin (A), the compound **2a** (B), and the compound **2b** (C). Applied concentrations were from 1, 10, and 100 µM. Data obtained from four experiments are presented as Mean ± S.D. * - Statistical significance compared to the untreated control; * - 0.01 $<p < 0.05$; ** - 0.001 $<p < 0.01$; *** - $p < 0.001$.

**Reference:**

1. Zhurakulov S.N, Narbutaeva D.A, Karakulova A.M, Tursunkhodzhaeva F.M, Vinogradova V.I. Aminomethylation of Quercetin by Tetrahydroisoquinoline Derivatives and Their Biological Activity. *Chemistry of Natural Compounds*. **2023**; *59*, 655-661.
